# Long-term use of non-steroidal anti-inflammatory drugs and the risk of myocardial infarction in the general population

**DOI:** 10.1186/1741-7015-3-17

**Published:** 2005-11-29

**Authors:** Luis A García Rodríguez, Antonio González-Pérez

**Affiliations:** 1 Centro Español de Investigación Farmacoepidemiológica (CEIFE), Madrid, Spain

## Abstract

**Background:**

Recent data indicate that chronic use of coxibs leads to an increased occurrence of thrombotic cardiovascular events. This raises the question as to whether traditional non-steroidal anti-inflammatory drugs (tNSAIDs) might also produce similar hazards. Our aim has been to evaluate the association between the chronic use of tNSAIDs and the risk of myocardial infarction (MI) in patients.

**Methods:**

We performed a nested case-control analysis with 4,975 cases of acute MI and 20,000 controls, frequency matched to cases by age, sex, and calendar year.

**Results:**

Overall, current use of tNSAID was not associated with an increased risk of MI (RR:1.07;95%CI: 0.95–1.21). However, we found that the relative risk (RR) of MI for durations of tNSAID treatment of >1 year was 1.21 (95% CI, 1.00–1.48). The corresponding RR was 1.34 (95% CI, 1.06–1.70) for non-fatal MI. The effect was independent from dose. The small risk associated with long-term use of tNSAIDs was observed among patients not taking low-dose aspirin (RR: 1.29; 95% CI, 1.01–1.65). The effect of long-term use for individual tNSAIDs ranged from a RR of 0.87 (95% CI, 0.47–1.62) with naproxen to 1.38 (95% CI, 1.00–1.90) with diclofenac.

**Conclusion:**

This study adds support to the hypothesis that chronic treatment with some tNSAIDs is associated with a small increased risk of non-fatal MI. Our data are consistent with a substantial variability in cardiovascular risks between individual tNSAIDs.

## Background

The recent withdrawal of rofecoxib together with new data showing that other cyclo-oxygenase-2 (COX-2) inhibitors might also be associated with an increased risk of developing adverse cardiovascular events, mainly acute myocardial infarction (MI), has created a climate of uncertainty surrounding the safety of these drugs and traditional non-steroidal anti-inflammatory drugs (tNSAIDs) [1,2]. In particular, this latter concern has been fostered by the preliminary communication of a press release suggesting that naproxen may be associated with an increased risk of cardiovascular and cerebrovascular events [3]. Two recently published studies found a similar result [4,5]. A feature in these newly released reports was that an increased risk only became apparent after prolonged administration of the suspected agent (in particular, coxibs), and notably in patients at a relatively low initial risk of cardiovascular disease.

Recently, we published a large epidemiologic study that evaluated the association between tNSAIDs as a class, as well as individual tNSAIDs, and the risk of MI [6]. Our overall estimate for tNSAIDs was compatible with either no association or a small increased risk, while the corresponding estimate for naproxen was compatible with either no association or a small reduced risk. We reported only briefly on the effect of duration of therapy in that paper, but now present more detailed information on the impact of duration of treatment on the relationship between tNSAIDs and MI.

## Methods

We have used the same data set and similar analytical models to those previously reported [6]. In brief, the design was of a prospective cohort study with nested case-control analysis. Overall 4,975 cases of acute MI and death from coronary heart disease (CHD) aged 50 to 84 years were identified between 1997 and 2000, using the UK General Practice Research Database. A total of 20,000 controls were randomly sampled and frequency matched to cases by age, sex, and calendar year. Both cases and controls were required to be enrolled with their general practitioner for at least two years before entering the study. Using this data set, we estimated the effect of duration of tNSAIDS as a class and 3 individual tNSAIDs (diclofenac, ibuprofen and naproxen) on the risk of MI. We computed estimates of odds ratios using unconditional logistic regression to estimate the relative risks (RR)[7], and 95% confidence interval (CI) of MI associated with current use of tNSAIDs compared to non-use. Estimates were adjusted for sex, age, calendar year, anemia, smoking status, alcohol use, diabetes, hypertension, hyperlipidemia, body mass index, rheumathoid arthritis, osteoarthritis, prior cardiovascular disease, use of steroids, anticoagulants, aspirin, and paracetamol. We performed several sensitivity analyses to see whether the observed effect was independent of variations in the operational definition of exposure as well as duration. In the main analysis, we identified NSAID prescriptions before the index date for cases and controls, and categorized exposure to NSAIDs as in the original publication: "current," when the supply of the most recent prescription lasted until index date or ended in the 30 days before the index date; "recent," when it ended between 31 and 180 days before the index date; "past," when it ended between 6 months and 2 years before the index date; and "non-use," when there was no recorded use in the 2 years before the index date. We repeated the same regression models using a more restrictive time-window for current use of 7 days prior to the index date. We studied the effect of duration among current users, and evaluated duration of use adding the periods of "consecutive" prescriptions, defined as an interval of <1 month (main analysis) between the end of supply of one prescription (assuming adherence) and the date of prescription of the subsequent one. In secondary analyses of duration, we varied the interval to be either <1 week or <2 months. Finally, we performed 2 additional analyses: first one on "new current users" (those current users who did not receive a prescription for an NSAID in the 6 months prior to starting on NSAIDs); and the second being past users of NSAIDs (patients with NSAID use that ended between 31 days and 2 years before the index date) as the reference group.

## Results

**Figure 1:**
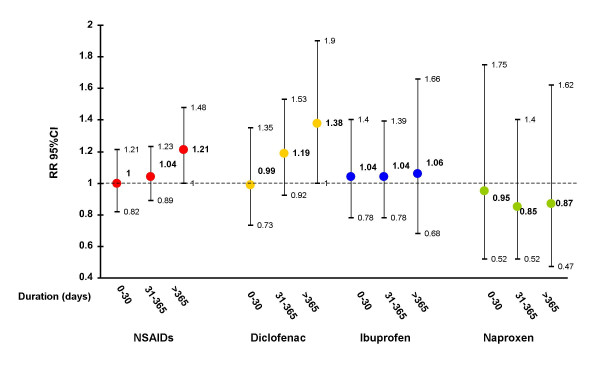
**Duration of use of NSAIDs and individual NSAIDs among current users (use within a month) and risk of MI. **Overall, current use of tNSAIDs was associated with a RR of 1.07 (95% CI, 0.95–1.21). The corresponding estimates for diclofenac, ibuprofen and naproxen were 1.17 (0.98–1.40), 1.05 (0.86–1.28) and 0.89 (0.64–1.25), respectively. Duration of use was computed adding the periods of "consecutive" prescriptions, defined as an interval of less than one month between the end of supply of one prescription and the date of prescription of the subsequent one. Estimates are adjusted for sex, age, calendar year, anemia, smoking status, alcohol use, diabetes, hypertension, hyperlipidemia, BMI, RA, OA, prior cardiovascular disease, use of steroids, anticoagulants, aspirin, paracetamol, and NSAIDs. The duration response trends for NSAIDs, diclofenac, ibuprofen and naproxen were P = .04, P = .02, P = .47, P = .54 respectively.

Overall, current intake of tNSAIDs was associated with a RR of 1.07 (95% CI, 0.95–1.21; Figure 1). The corresponding estimate when defining current exposure as use within a week prior to index date was 1.02 (95% CI, 0.90–1.17). The risk for treatment duration <1 year was no different from non-use, and for treatment duration >1 year, the RR was 1.21 (95% CI, 1.00–1.48): test for duration response trend, p .04. This estimate was slightly increased when using past users of NSAID as the reference group (RR:1.34;95%CI: 1.10–1.64). The median duration of use among long-term users was 2.8 years among cases and 2.6 among controls. A similar pattern of small increased risk of MI with longer duration was present with diclofenac, while this trend was not apparent for ibuprofen. On the other hand, the estimate of current use of naproxen was compatible with either no association or a small reduced risk of MI. The effect was present after a treatment of 1 month and persisted over longer durations of use (RR: 0.86; 95% CI, 0.58–1.27). Estimates of duration effects for tNSAIDs overall and naproxen, in particular, with 3 different assumptions about intervals between tNSAID use applied in the definition of the duration variables (Table 1). The increased risk with long-term treatment of tNSAIDs was most apparent when gaps between consecutive prescriptions were not permitted to exceed 7 days (RR: 1.31; 95% CI, 0.94–1.81). The varying intervals had no major impact on the estimates of duration-response associated with naproxen. We performed a similar sensitivity analysis for exposure to inhaled steroids (a priori not associated with MI). The estimates were similar in the 3 different scenarios (in order of increasing laxitude in the gap definition: 0.97 (0.62–1.49);0.95 (0.72–1.26);1.12 (0.91–1.37)). The duration effect of NSAIDs according to the concomitant use of low-dose aspirin and daily dose of tNSAIDs is shown in Table 2. The excess risk associated with long term use of tNSAIDs was observed among patients not taking concomitantly low-dose aspirin (RR: 1.29; 95% CI, 1.01–1.65), while long term use of tNSAIDs did not appear to increase the risk of MI among patients taking cardioprotective aspirin (RR: 0.90; 95% CI, 0.61–1.32). Data were too scarce to evaluate the interaction between aspirin use and chronic treatment with individual NSAIDs. No major variation in the risk of MI associated with chronic use of tNSAIDs was observed between users of low-medium dose versus high dose (Table 2). The corresponding estimates of RR associated with NSAID duration of >1 year for fatal and non-fatal MI were 1.02 (95% CI, 0.76–1.37) and 1.34 (95% CI, 1.06–1.70), respectively. The corresponding estimates of non-fatal MI for diclofenac, ibuprofen, and naproxen were 1.82 (1.27–2.62), 1.19 (0.70–2.03), and 1.00 (0.48–2.10), respectively. "New current users" with duration of use longer than 1 year had a RR of 1.31 (95%CI: 0.95–1.80).

**Table 1:** Duration of use of NSAIDs and naproxen among current users (use within a month) and risk of MI according to different definitions of the interval between consecutive prescriptions*

	Interval = 7 days	Interval = 30 days	Interval = 60 days
	OR (95%CI) †	OR (95%CI) †	OR (95%CI) †
NSAIDs			
Dura 0–30 days	0.97 (0.83–1.14)	1.00 (0.82–1.21)	1.13 (0.92–1.40)
Dura 31–365 days	1.14 (0.97–1.34)	1.04 (0.89–1.23)	1.00 (0.83–1.19)
Dura >365 days	1.31 (0.94–1.81)	1.21 (1.00–1.48)	1.10 (0.93–1.30)
			
Naproxen‡			
Dura 0–30 days	0.81 (0.49–1.35)	0.95 (0.52–1.75)	0.91 (0.46–1.79)
Dura > 30 days	0.94 (0.61–1.45)	0.86 (0.58–1.27)	0.88 (0.60–1.28)

* Duration of use was computed adding the periods of "consecutive" prescriptions, defined as varying intervals between the end of supply of one prescription and the date of prescription of the subsequent one.

† Estimates are adjusted for sex, age, calendar year, anemia, smoking status, alcohol use, diabetes, hypertension, hyperlipidemia, BMI, RA, OA, prior cardiovascular disease, use of steroids, anticoagulants, aspirin, paracetamol, and NSAIDs.

‡ Due to the limited number of observations in the duration strata of 31–365 days and the lack of heterogeneity in estimates of risk between this strata and the duration strata of greater than one year, we collapsed the two of them into one single duration strata.

**Table 2:** Duration of NSAID use among current users (use within a month) and risk of MI stratified by aspirin use and NSAID daily dose*

	Cases (%)	Controls (%)	OR (95%CI)
Aspirin non users†			
NSAID duration			
Dura 0–30 days	100 (28.2)	481 (31.2)	1.03 (0.81–1.30)
Dura 31–365 days	149 (42.0)	680 (44.1)	1.04 (0.85–1.27)
Dura >365 days	106 (29.8)	381 (24.7)	1.29 (1.01–1.65)
			
Aspirin current users†			
			
NSAID duration			
			
Dura 0–30 days	46 (26.4)	91 (28.8)	0.94 (0.64–1.40)
Dura 31–365 days	77 (44.3)	126 (39.9)	1.08 (0.78–1.50)
Dura >365 days	51 (29.3)	99 (31.3)	0.90 (0.61–1.32)

NSAID low-medium dose‡			
NSAID duration			
Dura 0–30 days	92 (28.5)	350 (31.3)	1.04 (0.81–1.34)
Dura 31–365 days	136 (42.1)	484 (43.4)	1.05 (0.84–1.32)
Dura >365 days	95 (29.4)	282 (25.3)	1.30 (1.00–1.71)
			
NSAID high dose‡			
NSAID duration			
Dura 0–30 days	70 (27.2)	269 (30.8)	0.98 (0.73–1.31)
Dura 31–365 days	110 (42.8)	382 (43.8)	1.10 (0.86–1.41)
Dura >365 days	77 (30.0)	222 (25.4)	1.21 (0.90–1.64)

* Duration of use was computed adding the periods of "consecutive" prescriptions, defined as an interval of less than one month between the end of supply of one prescription and the date of prescription of the subsequent one.

† Estimates are adjusted for sex, age, calendar year, anemia, smoking status, alcohol use, diabetes, hypertension, hyperlipidemia, BMI, RA, OA, prior cardiovascular disease, use of steroids, anticoagulants and paracetamol.

‡ Estimates are adjusted for sex, age, calendar year, anemia, smoking status, alcohol use, diabetes, hypertension, hyperlipidemia, BMI, RA, OA, prior cardiovascular disease, use of steroids, anticoagulants, aspirin and paracetamol. Cut-off values for dose in mg were: aceclofenac 100, acemetacin 120, apazone 600, diclofenac 75, etodolac 400, fenbufen 900, fenoprofen 1200, flurbiprofen 150, ibuprofen 1200, indomethacin 75, ketoprofen 100, mefenamic 1000, meloxicam 7.5, nabumetone 1000, naproxen 500, piroxicam 10, sulindac 200, tenoxicam 10, and tiaprofenic acid 450.

## Discussion

This additional analysis shows that use of tNSAIDs as a class for duration of continuous use of <1 year in general practice appears not to have a clinically relevant influence on the occurrence of MI. Our data are also compatible with the possibility that long-term therapy with tNSAIDs may confer a small excess risk of developing MI of ~20%, in particular of non-fatal MI. Our risk estimate is similar to the one provided by the authors of the first published epidemiologic study that analyzed the association between tNSAIDs and MI, who reported an estimate of relative risk for tNSAID duration longer than one year of 1.25; (95% CI, 0.90–1.72)[8]. The excess risk of MI among chronic users appears to be independent of NSAID daily dose and concentrated among patients not taking low-dose cardioprotective aspirin (eg. patients on average with a low cardiovascular profile). Our sensitivity analysis on the impact of varying operational definitions of duration for tNSAIDs as a class yielded small differences in the corresponding estimates associated with long-term treatment. The most stringent criteria of requiring a gap or interval of no greater than 1 week were the circumstances under which the increased risk with long-term tNSAID therapy was most noticeable, whereas the most liberal gap (up to 2 months) revealed a relative risk closer to no increased risk. This last result could be due in part to misclassification of the "true" continuous duration sequence in real life.

Even though our study was not designed to make direct comparissons between NSAIDs, we observed that at least three individual NSAIDs (the 3 most widely used) appeared to have different risk profile within a seemingly heterogeneous group of tNSAIDS. A clear duration-response was found for diclofenac, with an emerging risk associated with chronic use extending over 1 year. Secondly, we found no clear trend with ibuprofen duration – albeit that could, in part, be explained by some misclassification of exposure due to over the counter use of ibuprofen. Finally, there was no evidence of an increased risk associated with naproxen use and our results are more compatible with a minor reduced risk of MI. Misclassification due to over the counter use could also be an issue for naproxen. The risk with naproxen was relatively insensitive to varying the interval used to calculate the length of treatment duration. A potential small protective effect associated with naproxen has been reported in most epidemiological studies [6], but not all [9]. These results are at odds with the unpublished suggestion of a cardiovascular hazard from naproxen in a clinical trial stopped prematurely last year and under controversial circumstances [3]. The apparent level of protection afforded by regular intake of naproxen in our study may be due to suppression of platelet thromboxane production, when administered twice a day [11]. This is the mechanism by which aspirin affords cardioprotection. However, unlike aspirin, some, but not all patients, treated chronically with naproxen maintain suppression of thromboxane throughout the whole dosing interval at a level compatible with effective platelet inhibition [10,11]. Furthermore, many individuals classified as "chronic users" of naproxen in this general population may not have been compliant with the required dosing regimen (twice a day) all throughout the period of prescribed drug intake further diluting the benefit of an "aspirin effect". Thus, one would not expect to observe a benefit similar to the one of low-dose aspirin in the range of a 20–25% reduction in the incidence of events. No other widely used tNSAID shares the same pharmacodynamic/pharmacokinetic properties of naproxen on platelets and consequently other tNSAIDs are not expected to confer any degree of cardioprotection. Due to scarce numbers of chronic users for other individual NSAIDs, we could not estimate their respective duration-response relationship. Yet, one can not assume a class effect based on the heterogeneity observed between the 3 individual NSAIDs. In particular, these observations question the emerging strategy of comparing naproxen with "non-naproxen tNSAIDs". Direct comparison of our findings with those of recent coxib/tNSAID randomized clinical trials (RCTs) are hampered by the use in RCTs of significantly higher average daily doses of tNSAIDs together with more strict compliance than the one occuring in "real life" situations [1-3].

We analyzed the risk of inhaled steroids (a drug class considered a priori not to be associated one way or the other with the risk of MI) to confirm the internal validity of our sensitivity analysis of the operational definition of duration. The estimates of RR for long-term duration of inhaled steroids changed minimally using the 3 different intervals, ranging between 1.0 and 1.1. A limitation in the interpretation of our results is the imprecision of most estimates of risk, given that the magnitude of the "true" association we have purported to evaluate is most likely weak, either of increased risk with long-term duration of NSAIDs or reduced risk with naproxen. In this scenario, the common limitations in all observational studies (potential residual or unmeasured confounding) could be affecting our results. Over-the-counter (OTC) medications are not recorded in our source of information. Yet, it should be noted that OTC long-term use of tNSAIDs or cardioprotective aspirin is uncommon in the UK and could have slightly underestimated our measures of association, in particular ibuprofen.

## Conclusion

The overall experience with tNSAIDs in our study is generally congruent with a neutral effect on cardiovascular disease although there is a suggestion of a small excess risk with chronic exposure, especially for non-fatal MI. The summary estimate for the tNSAID group is composed of substantial variation in risk between the three studied individual tNSAIDs suggesting a biologically plausible heterogenity in cardiovascular risk [1,12]. Our study suggests either no effect or a small reduction of cardiovascular risk during sustained treatment with naproxen, a small increased risk with diclofenac, and an undetectable risk with ibuprofen. Larger observational studies and, if at all possible, randomised clinical trials will be necessary to address this hypothesis of mechanistic heterogenity amongst tNSAIDs with respect to cardiovascular risk.

## Abbreviations

cyclo-oxygenase-2 :COX-2

myocardial infarction: MI

traditional non-steroidal anti-inflammatory drugs : tNSAIDs

coronary heart disease: CHD

relative risk: RR

randomized clinical trials: RCTs

over-the-counter: OTC

## Competing interests

We used data from a previous study that was supported in part by a research grant from Pharmacia. Two employees of Pharmacia (now Pfizer) coauthored that paper. In the present study the funding body had no role.

## Authors' contributions

Both LAGR and AGP participated in the conception, design, analysis, interpretation of data, and drafting of the manuscript. Both authors read and approved the final manuscript.

## Pre-publication history

The pre-publication history for this paper can be accessed here:


